# *Aeromonas hydrophila*, an Emerging Causative Agent of Freshwater-Farmed Whiteleg shrimp *Litopenaeus vannamei*

**DOI:** 10.3390/microorganisms7100450

**Published:** 2019-10-14

**Authors:** Huihua Zhou, Chunlei Gai, Guifang Ye, Jian An, Kai Liu, La Xu, Haipeng Cao

**Affiliations:** 1 National Pathogen Collection Center for Aquatic Animals, Shanghai Collaborative Innovation for Aquatic Animal Genetics and Breeding, Shanghai Engineering Research Center of Aquaculture, Shanghai Ocean University, Shanghai 201306, China; zhouHH827@163.com (H.Z.); 18621092058@139.com (G.Y.); 2 Marine Biology Institute of Shandong, Qingdao 266104, China; chunlei317@163.com (C.G.); 18561729912@163.com (L.X.); 3 Lianyungang Marine and Fisheries Development Promotion Center, Lianyungang 222000, China; anjian520@sohu.com; 4 Institute of Fishery Science, Hangzhou Academy of Agricultural Sciences, Hangzhou 310024, China; liukai0106@hz.cn

**Keywords:** *Aeromonas hydrophila*, *Litopenaeus vannamei*, *Punica granatum*, florfenicol, synergist

## Abstract

*Aeromonas hydrophila* is a well-known bacterial pathogen associated with mass mortalities in aquaculture. Yet, few reports are available on whiteleg shrimp-pathogenic *A. hydrophila*. In the present study, a virulent isolate WS05 was confirmed as a causative agent of diseased freshwater-cultured whiteleg shrimp and showed a mean lethal dose (LD_50_) value of 4.8 × 10^4^ CFU mL^−1^. It was identified phenotypically and molecularly as an *A. hydrophila* strain, and exhibited susceptibility to several veterinary antibiotics extensively used in aquaculture, including cotrimoxazole, doxycycline, florfenicol, neomycin, and tetracycline. In view of the strongest inhibition zone of florfenicol against isolate WS05, the synergistic effect of the combinations of florfenicol and herb extracts was further evaluated, and the result indicated that *Punica granatum* extract was a potential synergist of florfenicol against isolate WS05 and the fractional inhibitory concentration index (FICI) for the florfenicol-*P. granatum* extract was calculated as 0.31. When combined with 7.81 mg mL^−1^ *P. granatum* extract, the minimum inhibitory concentration (MIC) of florfenicol against isolate WS05 was reduced from 0.50 to 0.03 mg L^−1^, and its activity against isolate WS05 was also enhanced with a significant reduction of ≥3.61 log in cell density after 24 h of treatment compared with that in the single drug treatment. In addition, the protective effect was potentiated by the combination of florfenicol and *P. granatum* extract, with a cumulative mortality of 36.66% (*p* < 0.05) and 33.33% (*p* < 0.05) lower than that in the single treatment with florfenicol and *P. granatum* extract after the challenge with isolate WS05 for seven days. As far as we know, this is the first study to describe whiteleg shrimp-pathogenic *A. hydrophila* and suggest *P. granatum* extract as a potential synergist of florfenicol against the *A. hydrophila* pathogen.

## 1. Introduction

The whiteleg shrimp *Litopenaeus vannamei* is one of the most important commercial shrimp species and is extensively cultivated in Central and South America, the United States of America (USA), East and South-East Asia, the Middle East, and Africa [1], which accounts for 75% of the global shrimp products [2]. Especially in China, with the rapid development of farming techniques, the whiteleg shrimp has been successfully cultured in freshwater since 2000 [3], with an annual freshwater aquaculture production of over 591,000 tons in 2017 [4]. However, this industry has been seriously affected by bacterial diseases [5], which are caused by several bacterial pathogens, such as *Vibrio parahaemolyticus*, *Vibrio harveyi*, *Vibrio cholerae*, *Proteus penneri*, *Aeromonas schubertii*, and *Shewanella algae* [6,7,8,9,10,11]. In order to prevent and control bacterial diseases, antibiotics are widely used in whiteleg shrimp aquaculture [12]. However, the widespread and frequent use of antibiotics in shrimp aquaculture has resulted in the development of antibiotic resistance among pathogens infecting cultured animals and humans [12]. Herbs with potential antibacterial activity against *Aeromonas hydrophila* have a crucial role in disease management in aquaculture [13]. Extracts from herbs, such as *Acorus calamus*, *Indigofera aspalathoides*, *Coleus aromaticus*, *Thymus vulgaris*, and *Trifolium pannonicum*, have been reported to effectively inhibit pathogenic *A. hydrophila* [14,15,16,17]. Many studies have also proved that oral administration of extracts from herbs, such as *Allium sativum*, *Epilobium hirsutum*, *Panax quinquefolium*, and *Toona sinensis*, can greatly enhance resistance against *A. hydrophila* infection in fish [18,19,20,21]. Therefore, in order to limit the use of antibiotics, the use of herb extracts in combination with conventional antibiotics is commonly suggested for the treatment of bacterial pathogens in aquaculture [22], which could significantly reduce the dosage of antibiotics against bacterial pathogens [23]. Jedlickova et al. (1992) found that the combinations of 1,8-cineol, linalool, and terpinen-4-ol with amikacin, gentamicin, and tobramycin could strongly inhibit the growth of *Escherichia coli* [24]; Jayaraman et al. (2010) showed that combinations of sulfamethoxazole and myricetin could significantly enhance antibacterial activities against *Pseudomonas aeruginosa* strains [25]. Yet, little has been documented on whiteleg shrimp-pathogenic *Aeromonas hydrophila* and its control with combinations of herb extracts and antibiotics.

In late May of 2019, a severe disease with the typical signs of hepatopancreatic shrinking occurred in nearly the whole shrimp farming regions of Fengxian, Shanghai, China, and resulted in a cumulative mortality of 50% to 100%. In the present study, we confirm that a virulent isolate of *A. hydrophila* was the causative agent of the disease in freshwater-cultured whiteleg shrimp, and its taxonomic position, virulence, and antibiotic susceptibility were examined. Furthermore, in view of the strongest inhibition zone of florfenicol against the isolate, the control of the isolate with combinations of florfenicol and herb extracts was further evaluated. To our knowledge, this is the first report of *A. hydrophila* as a bacterial pathogen of freshwater-cultured whiteleg shrimp, and the findings of this study can be used as a reference for disease control and health management in shrimp aquaculture.

## 2. Materials and Methods

### 2.1. Shrimp and Reagents

Twenty moribund freshwater-cultured whiteleg shrimp (6.80 ± 0.05 g in weight) suffering from hepatopancreatic shrinking were sampled from an infected freshwater pond of a shrimp farm in Fengxian, Shanghai, China during May 2019 and were placed into sterile bags and kept on ice during the 1-h transport to the laboratory as recommended by Jayasinghe et al. (2008) [26]. In total, 340 healthy shrimp (5.26 ± 0.36 g) for the infection assay were obtained from unaffected ponds of a shrimp farm in Lianyungang Aquaculture Co., Ltd., Jiangsu, China and maintained in 34 glass aquaria (76 cm × 50 cm × 48 cm) (10 shrimp per aquarium) with 100 L aerated filtered farm water as recommended by Moss et al. (1992, 2001) [27,28] with an initial pH of 7.50, 6.2 mg L^−1^ of dissolved oxygen, 0.11 mg L^−1^ of total ammonia, and 0.01 mg L^−1^ of nitrite at 28 °C for 14 days. Next, 240 healthy shrimp (0.62 ± 0.11 g) for the protective effect assay were obtained from unaffected ponds of a shrimp farm in Rudong Aquaculture Co., Ltd., Jiangsu, China, and maintained in 24 glass aquaria (76 cm × 50 cm × 48 cm) (10 shrimp per aquarium) supplied with 100 L aerated filtered farm water with an initial pH of 7.64, 6.6 mg L^−1^ of dissolved oxygen, 0.10 mg L^−1^ of total ammonia, and 0.01 mg L^−1^ of nitrite at 28 °C for 14 days. Their health status was assessed through a careful examination of the external appearance, gut condition, growth situation, physical behavior, and feeding trends as recommended by the Marine Products Export Development Authority and the Network of Aquaculture Centers in Asia-Pacific (2003) [29], as well as by culturing hemolymph and hepatopancreas smears from a few sampled animals on nutrient agar (NA) plates (Sinopharm Chemical Reagent Co., Ltd., Shanghai, China) for the absence of any bacterial pathogens as recommended by Biswas et al. (2012) [30]. Filtered farm water used in our laboratory was prepared by passing 6 m^3^ of water, which was obtained from the natural Luchaoyin River, successively through 15-denier-size polyester fiber and a 60-pore per inch (ppi) polyurethane sponge in a water-filtration device (Shanghai Haisheng Biotech. Co., Ltd., Shanghai, China), with a flow rate of 0.24 m^3^ min^−1^ and a circulation rate of 8 times d^−1^ as recommended by Luo et al. (2008) [31], and was sterilized at 121 °C for 20 min to remove bacteria prior to its use. Florfenicol (98%) was purchased from Tianchen Biotech. Co. Ltd., Wuhan, China. Herbs were obtained from Marine Biology Institute of Shandong, Shandong, China. Reagents were of analytical grade from Sinopharm Chemical Reagent Co., Ltd., Shanghai, China.

### 2.2. Confirmation of the Causative Pathogen

Each sampled diseased whiteleg shrimp was disinfected externally with 75% alcohol and dissected in the laboratory according to Cao et al. (2014) [9]. To verify the potential pathogens, a squash of organs (gill, hepatopancreas, muscle, intestine) were made and carefully examined for parasites under the microscope (YS100, Nikon, Tokyo, Japan) as described by Ekanem et al. (2011) [32]. Meanwhile, the virological examination was also conducted as described by Pan et al. (2009) [33]. Briefly, the homogenate of organs (gill, hepatopancreas, muscle, intestine) was made and filtered through 0.22-μm-pore-size membrane filter to remove bacteria. Two aquaria of 10 healthy shrimp were injected muscularly with 0.1 mL of each bacteria-free organ filtrate. Another two aquaria of 10 healthy shrimp, which were exposed to the same experimental conditions and injected muscularly with 0.1 mL of normal saline, served as the control. Experimental shrimp were kept at 28 °C without water change. The mortality and any visible changes of the experimental shrimp were recorded every day for 15 days. In addition, samples from organs (hepatopancreas, muscle) of each diseased shrimp were cut and directly streaked onto NA plates as recommended by Cao et al. (2018) [34]. After incubation for 24 h at 28 °C, the dominant uniform isolates were purified by streaking and re-streaking onto NA plates (Sinopharm Chemical Reagent Co., Ltd., Shanghai, China). Only the dominant isolates with dense virtually pure culture growth on NA plates were obtained as recommended by Orozova et al. (2012) [35] and Cao et al. (2014) [9] After being initially identified by 16S rRNA gene sequencing as recommended by Petti et al. (2005) [36], the pure isolates were further inoculated onto an NA plate, incubated at 28 °C for 24 h, and washed with sterile normal saline into a sterile tube. Their cell densities were determined by counting the colony forming units (CFU) on NA plates after a 10-fold serial dilution in sterile normal saline. Induced infection of the pure isolates was performed according to Cao et al. (2015) [10]. Briefly, two aquaria of 10 healthy shrimp were challenged by immersion in 100 L of water containing the isolates at a cell density of 5.0 × 10^6^ CFU mL^−1^. Another two aquaria of 10 healthy shrimp, which were exposed to the same experimental conditions and remained unchallenged, served as the control. Experimental shrimp were kept at 28 °C without water change. The mortality and any visible changes of the experimental shrimp were recorded every day for 7 days. Dead shrimp were immediately removed and the hepatopancreas sampled on NA plates to confirm if the mortality was caused by the challenge isolate. Besides, ultrathin sections were prepared from hepatopancreas of the infected and healthy shrimp for light and electron microscopic examination as described by Zhou et al. (2012) [7]. Briefly, for light microscopy, the hepatopancreas was fixed in Boun’s solution, followed by dehydration in a graded series of alcohol (75%, 85%, 90%, 95%, 100%) (30 min in each dehydrating agent), cleared by xylol, embedded with paraffin, stained with hematoxylin and eosin, and then observed under a light microscope (Eclipse E100, Nikon, Tokyo, Japan). For transmission electron microscopy, the hepatopancreas was first fixed in 2.5% glutaraldehyde in 0.1 M PBS (pH 7.4) for 2 h at 4 °C, followed by post-fixation in 1% osmium tetroxide for 2 h, dehydrated in a graded series of alcohol (50%, 70%, 80%, 90%, 95%, 100%) and 100% acetone (15 min in each dehydrating agent), embedded in Spurr’s resin, stained with uranyl acetate and lead citrate, and then observed under a transmission electron microscope (HT770, Hitachi, Tokyo, Japan).

### 2.3. Identification of the Causative Pathogen

#### 2.3.1. Molecular Identification

The genomic DNA was extracted from the pathogenic isolate using the TIANamp DNA Kit (Tiangen Biotech. Co., Ltd., Beijing, China). The 16S rRNA, *gyrB*, and *rpoB* genes from the pathogenic isolate were amplified by PCR according to Cao et al. (2010) [37], Yáňez et al. (2003) [38], and Küpfer et al. (2006) [39], and sequenced by an ABI 3730 XL DNA Sequencer (Applied Biosystems, Waltham, MA, USA). Homology searches for 16S rRNA, *gyrB*, and *rpoB* gene sequences were performed using the Basic Local Alignment Search Tool version 4 (BLAST) software (National Library of Medicine, Bethesda, MD, USA) available at the National Center for Biotechnology Information (NCBI). Sequence alignment was carried out with Molecular Evolutionary Genetics Analysis version 5 (MEGA5) software (Arizona State University, Tempe, AZ, USA), and the phylogenetic trees were constructed using the neighbor-joining method.

#### 2.3.2. Phenotypic Identification

The phenotypic identification of the pathogenic isolate was performed by the API 20E system (Biomerieux, Lyon, France) as recommended by Abeyta et al. (2019) [40]. Briefly, the pathogenic isolate streaked on NA plates was incubated at 28 °C for 24 h, and then inoculated into the API 20E test strips (Biomerieux, Lyon, France) according to the instructions of the manufacturer. The test strips containing the isolate were incubated at 37 °C for 18 h and observed for biochemical reactions. The phenotypic characterization of *A. hydrophila* previously reported by Long et al. (2016) [41] and Ye et al. (2018) [42] served as a reference.

### 2.4. Bacterial Virulence Experiment

The bacterial virulence experiment consisted of one control and five treatment groups. Prior to this experiment, the pathogenic isolate was inoculated onto an NA plate, incubated at 28 °C for 24 h, and washed with sterile normal saline into a sterile tube. Its cell density was determined by counting the colony-forming units (CFU) on NA plates after a 10-fold serial dilution in sterile normal saline. Each group contained two glass aquaria (10 shrimp per aquarium) with 100 L aerated filtered farm water. In the treatment groups, the experimental shrimp in the glass aquaria were challenged by immersion with the pathogenic isolate at final cell densities of 2.0 × 10^3^ to 2.0 × 10^7^ CFU mL^−1^ in water as recommended by Teng et al. (2017) [43]. Another two aquaria of healthy shrimp, which were exposed to the same experimental conditions and remained unchallenged, served as the control. All of the test shrimp were kept at 28 °C without any water change. The mortality was recorded every day for 7 days. Dead shrimp were immediately removed and the hepatopancreas sampled on NA plates to confirm whether the mortality was caused by the challenge isolate. The mean lethal dose (LD_50_) value was calculated using the graphical probit method [44].

### 2.5. Antibiotics Susceptibility Assay

The susceptibility of the pathogenic isolate to veterinary antibiotics was examined using the Kirby–Bauer disk diffusion method described by Jones et al. (2001) [45] on NA plates. The susceptibility was determined by measuring inhibition zones after incubation at 28 °C for 24 h and was evaluated according to the guidelines of the manufacturer (Hangzhou Binhe Microorganism Reagent Co., Ltd., Hangzhou, China) as recommended by Cao et al. (2018) [34].

### 2.6. Synergistic Effect of Florfenicol–Herb Extracts Assay

Because of the strongest inhibition zone of florfenicol against the pathogenic isolate as shown in antibiotics susceptibility assay, florfenicol was chosen for further study. The synergistic effect of florfenicol in combination with herb extracts on the pathogenic isolate was evaluated in a 24-well microplate using the checkerboard method described by Chen et al. (2014) [46]. Prior to this assay, 1000 mg L^−1^ of florfenicol was prepared according to Liu et al. (2011) [47]. Extracts from peels of pomegranate *Punica granatum*, fruits of *Prunus mume* and *Fructus toosendan*, leaves of *Artemisia argyi*, *Polygonum aviculare*, *Cephalanoplos segetum*, and *Artemisia capillaries* and roots of the other herbs were prepared with distilled water as recommended by Rattanachaikunsopon and Phumkhachorn (2009) [48]. Briefly, each herb was oven-dried at 80 °C for 72 h, finely ground in a mortar, and extracted with the distilled water at a ratio of 1:10 (*w*/*v*) using a full-automatic herb-decocting kettle (Guangzhou Haizhu Electric Co., Ltd., Guangzhou, China). The mixture was then centrifuged at 13,000 rpm for 10 min at room temperature and the supernatant was collected as the herb extract. The concentration of each herb extract for each assay was adjusted to 1000 mg (dry weight) mL^−1^. Immediately after the pathogenic isolate was added to each plate well to a final cell density of 5 × 10^5^ CFU mL^−1^ as recommended by Liu et al. (2000) [49], antimicrobial agents were loaded per well with serial concentrations ranging from 0.125 to 512 mg L^−1^ for florfenicol and from 1.95 to 500 mg mL^−1^ for the herb extracts. The plates were incubated at 28 °C for 24 h. The minimum inhibitory concentrations (MICs) of the antimicrobial agents alone or in combination were determined after 24 h of incubation, which were defined as the lowest concentration showing no color change that exhibited complete inhibition of growth according to Haroun and Al-Kayali (2016) [50]. The fractional inhibitory concentration index (FICI) was calculated according to the formula as recommended by Liu et al. (2017) [51]: FICI = MIC (florfenicol in combination)/MIC (florfenicol alone) + MIC (herb extract in combination)/MIC (herb extract alone). The joint effect was evaluated according to the following criteria as suggested by Gong et al. (2005) [52]: Synergistic effect (FICI ≤ 0.5); additive effect (0.5 < FICI ≤ 1), no interaction (1 < FICI < 4) and antagonistic effect (FICI ≥ 4).

### 2.7. The in Vitro Activity of Florfenicol-Herb Extracts Assay

The in vitro activity of florfenicol in combination with the herb extract with a synergistic effect was examined by the time-kill curve method as recommended by Dong et al. (2016) [23]. The assay was carried out in 50-mL glass flasks and consisted of one control and three treatment groups (three flasks per group). Immediately after the pathogenic isolate was inoculated in 20 mL of autoclaved nutrient broth to a final cell density of 5.0 × 10^5^ CFU mL^−1^ as recommended by Liu et al. (2000) [49], antimicrobials were independently inoculated into autoclaved nutrient broth in the treatment groups to final concentrations of 0.03 mg L^−1^ florfenicol, 7.81 mg mL^−1^ *P. granatum* extract, and 0.03 mg L^−1^ florfenicol plus 7.81 mg mL^−1^ *P. granatum* extract, which were determined by the synergistic effect assay above. All of the mixtures were then incubated at 28 °C for 48 h as recommended by Dong et al. (2016) [23]. In the control group, only the pathogenic isolate was inoculated in autoclaved nutrient broth to a final cell density of 5.0 × 10^5^ CFU mL^−1^ and incubated as mentioned above. The cell density of the pathogenic isolate was measured every 12 h by spread plate counts on the NA plate [23,37].

### 2.8. Protective Effect of Florfenicol-Herb Extracts Assay

The protective effect assay consisted of one blank control, one negative control, three positive control, and three treatment groups. Prior to this assay, the pathogenic isolate was inoculated onto an NA plate, incubated at 28 °C for 24 h, and washed with sterile normal saline into a sterile tube. Its cell density was determined by counting the CFU on NA plates after a 10-fold serial dilution in sterile normal saline. Each group contained three glass aquaria (10 shrimp per aquarium) with 100 L aerated filtered farm water. In the treatment groups, antimicrobials were independently inoculated into the aerated filtered farm water in the aquaria to final concentrations of 0.03 mg L^−1^ florfenicol, 7.81 mg mL^−1^ *P. granatum* extract, and 0.03 mg L^−1^ florfenicol plus 7.81 mg mL^−1^ *P. granatum* extract, which were determined by the synergistic effect assay above. Thereafter, all of the shrimp were challenged by immersion through continuous exposure to the pathogenic isolate at a final cell density of 2.0 × 10^6^ CFU mL^−1^ in water as determined above. In the positive control groups, the shrimp were only treated with the antimicrobials. In the negative control group, the shrimp were only challenged by immersion with the pathogenic isolate at a final cell density of 2.0 × 10^6^ CFU mL^−1^ in water as described above. Another three aquaria of healthy shrimp, which were exposed to the same experimental conditions and remained unchallenged, served as the blank control. All of the test shrimp were kept at 28 °C without water change. The mortality was recorded every day for 7 days. Dead shrimp were immediately removed and the hepatopancreas sampled on NA plates to confirm if the mortality was caused by the challenge isolate. 

### 2.9. Statistical Analysis 

Statistical analysis was carried out using the statistical software SPSS 15.0 (SPSS, Inc.) to observe the difference in each assay. All of the data are presented as the mean ± standard deviation (SD) for the indicated number of each assay. Differences were considered statistically significant at *p* < 0.05 using analysis of variance according to Duncan’s test.

### 2.10. Ethics Statements

The experimental protocol strictly followed the guidelines for the ethical review of animal welfare and the general requirements for animal experiments, China. The present experiment was approved by the Institutional Animal Ethics Committee of Shanghai Ocean University (establishment date: 18 February 2016) with the permission No. 20171025 valid until 31 December 2020.

## 3. Results

### 3.1. Confirmation of the Causative Pathogen

**Figure 1:**
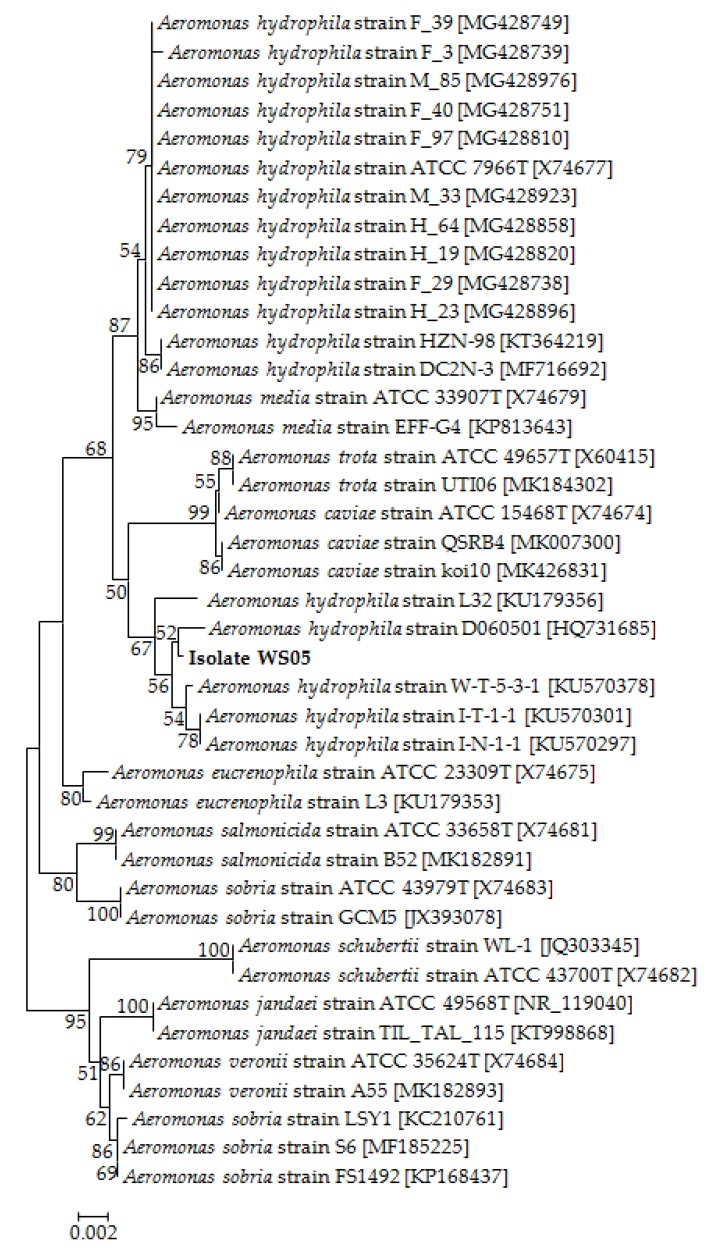
The 16S rRNA phylogenetic tree of 40 known bacteria and isolate WS05 constructed using the neighbor-joining method. The length of aligned sequences is 1430 bp. The bootstrap values (%) are shown besides the clades, accession numbers are indicated beside the name of strains, and scale bars represent distance values.

**Figure 2:**
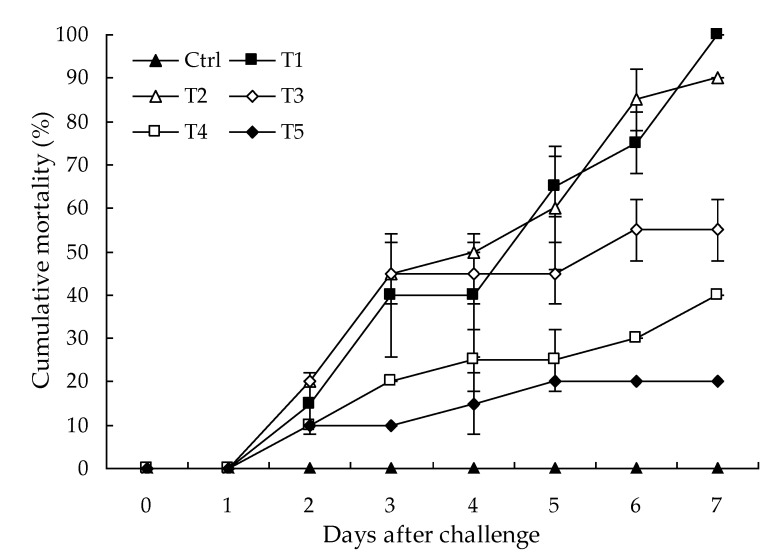
Virulence of isolate WS05 at final cell densities of 2.0 × 10^3^ to 2.0 × 10^7^ CFU mL^−1^ for experimental whiteleg shrimp. Ctrl, Control group; T1, Treatment with isolate WS05 at 2.0 × 10^7^ CFU mL^−1^; T2, Treatment with isolate WS05 at 2.0 × 10^6^ CFU mL^−1^; T3, Treatment with isolate WS05 at 2.0 × 10^5^ CFU mL^−1^; T4, Treatment with isolate WS05 at 2.0 × 10^4^ CFU mL^−1^; T5, Treatment with isolate WS05 at 2.0 × 10^3^ CFU mL^−1^. Data are presented as the mean ± SD.

**Figure 3:**
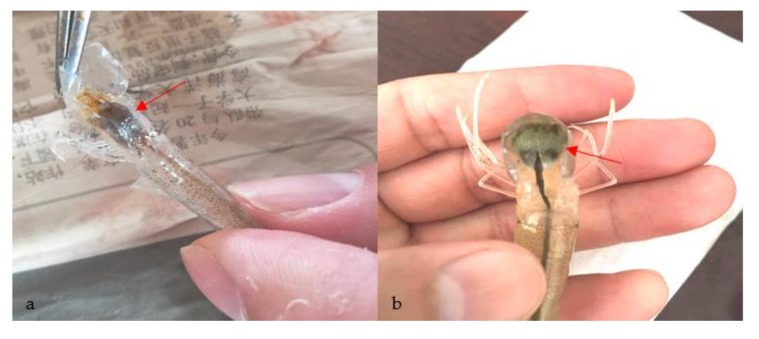
Gross signs of affected shrimp in the disease outbreak region. (**a**) Diseased shrimp. Arrow shows the atrophic hepatopancreas; (**b**) healthy shrimp. Arrow shows the normal hepatopancreas.

**Figure 4:**
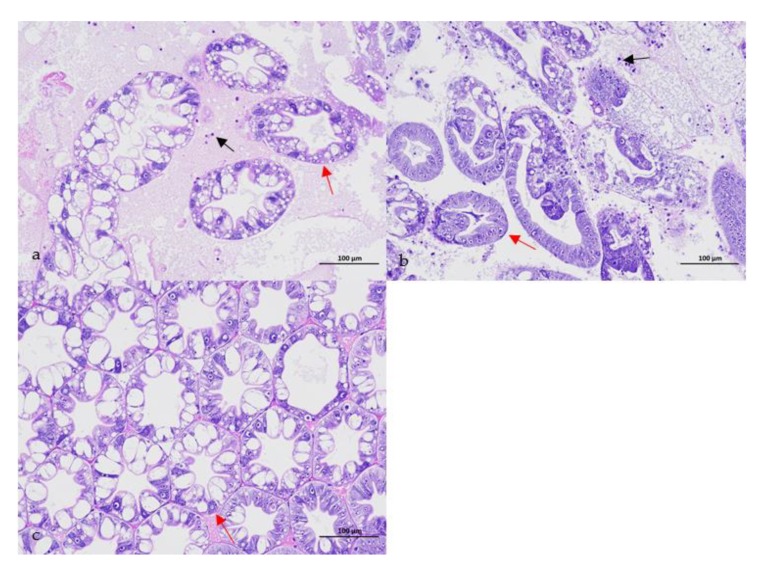
Histopathological changes in the atrophic hepatopancreas of affected shrimp. (**a**) Disordered arrangement of hepatopancreatic tubules (red arrow) and inflammatory cell infiltration (black arrow) in the naturally infected hepatopancreas; (**b**) disordered arrangement of hepatopancreatic tubules (red arrow) and inflammatory cell infiltration (black arrow) in the artificially infected hepatopancreas; (**c**) normal arrangement of hepatopancreas tubules (red arrow) in healthy hepatopancreas.

**Figure 5:**
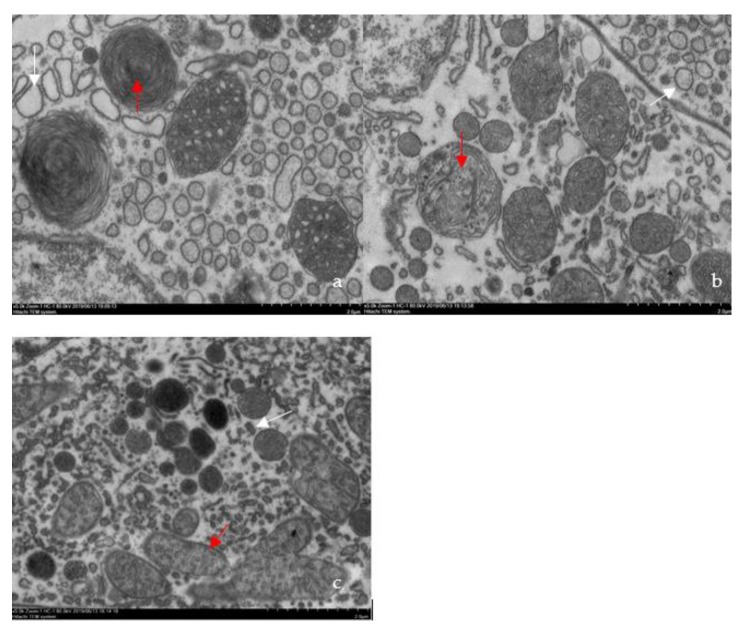
Ultrastructural changes in the atrophic hepatopancreas of affected shrimp. (**a**) Mitochondrial myelin body formation (red arrow) and dilatational endoplasmic reticulum (white arrow) in the naturally infected cell cytoplasm (5000 ×); (**b**) mitochondrial myelin body (red arrow), which is forming, and dilatational endoplasmic reticulum (white arrow) in the artificially infected cell cytoplasm (5000 ×); (**c**) normal mitochondria (red arrow) and endoplasmic reticulum (white arrow) in healthy cell cytoplasm (5000 ×).

No parasites were found in the diseased whiteleg shrimp, and all of the experimental shrimp challenged with the bacteria-free organ filtrate survived with no visible changes (data not shown), indicating that the disease was not caused by parasites or viruses. Five dominant isolates (numbered from WS01 to WS05) were recovered from the hepatopancreas of the diseased shrimp, which were identified as the members of the genus *Bacillus* and *Aeromonas* based on the analysis of their 16S rRNA genes (GenBank accession nos. MN367958, MN371228, MN367959, MN367960, MN148709) (Figure S1 and Figure 1), and none were isolated from the muscles. The result of the bacterial challenge in shrimp indicated that only the isolate (WS05) was pathogenic to whiteleg shrimp, showing an LD_50_ value of 4.8 × 10^4^ CFU mL^−1^ (Figure 2). In contrast, no visible changes or death were recorded in the control shrimp. The artificially infected shrimp exhibited the sign of hepatopancreas shrinkage similar to the naturally infected shrimp (Figure 3), and the same strain (WS05) was re-isolated from the experimentally diseased shrimp and confirmed by phenotypical and molecular identification. Furthermore, the histopathological changes in the hepatopancreas from naturally infected and artificially infected shrimp showed a disordered arrangement of hepatopancreatic tubules and inflammatory cell infiltration (Figure 4). Moreover, an electron microscopic analysis further showed the hepatopancreatic cell was damaged in both naturally infected and artificially infected shrimp, including abnormal formation of the mitochondrial myelin body and dilatational endoplasmic reticulum (Figure 5). These findings demonstrated that isolate WS05 was the causative agent of this disease.

### 3.2. Identification of the Pathogenic Isolate

**Figure 6:**
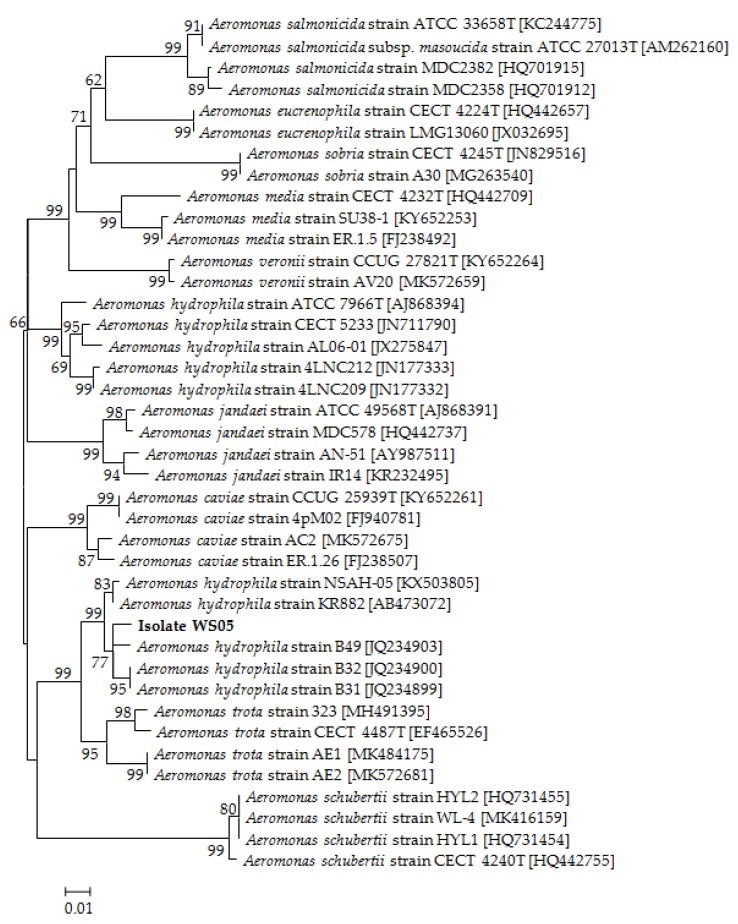
The phylogenetic tree based on *gyrB* gene sequences of 39 *Aeromonas* strains and isolate WS05 constructed using the neighbor-joining method. The length of aligned sequences is 765 bp. The bootstrap values (%) are shown besides the clades, accession numbers are indicated beside the name of strains, and scale bars represent distance values.

**Figure 7:**
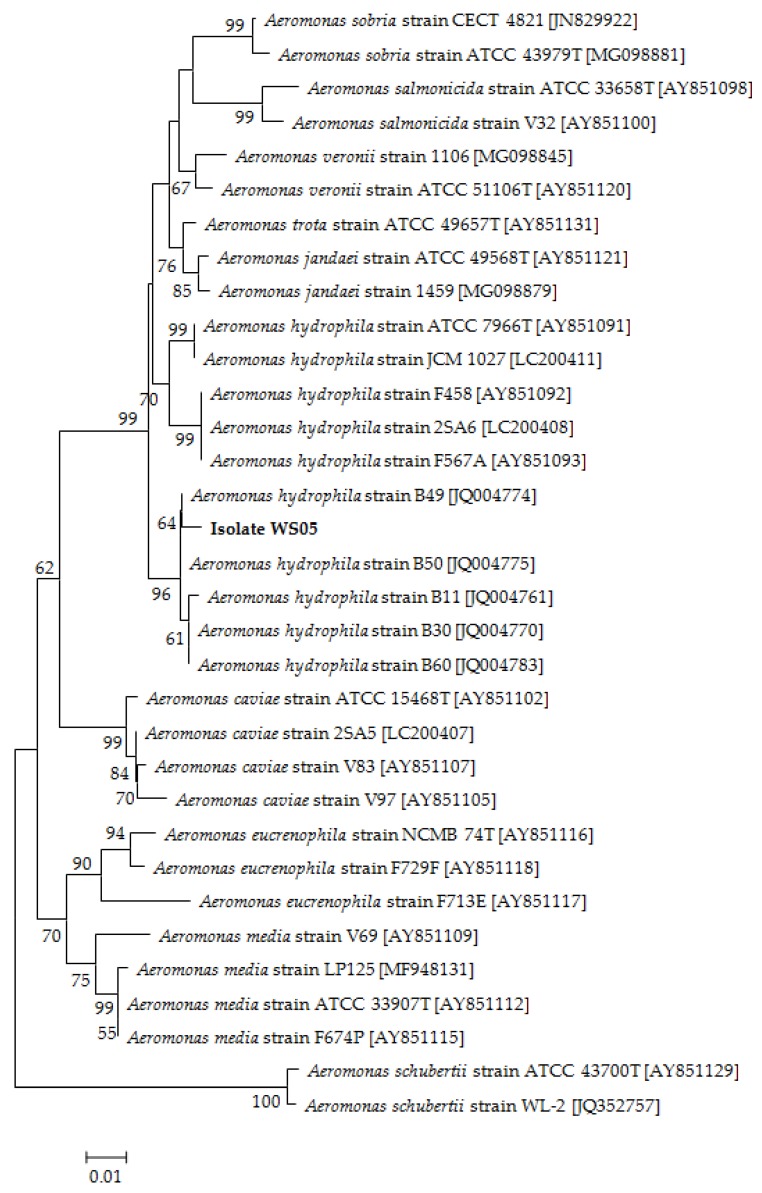
The phylogenetic tree based on *rpoB* gene sequences of 32 *Aeromonas* strains and isolate WS05 constructed using the neighbor-joining method. The length of aligned sequences is 423 bp. The bootstrap values (%) are shown besides the clades, accession numbers are indicated beside the name of strains, and scale bars represent distance values.

Isolate WS05 was identified phenotypically and molecularly as *A. hydrophila*. It exhibited the same phenotypic traits with the reference strains of *A. hydrophila* [41,42]. Isolate WS05 was positive for utilization of arginine, lysine, citrate, and tryptophan, and was negative for growth on amygdalin, inositol, melibiose, rhamnose, and sorbitol (Table 1). Furthermore, the 16S rRNA, *gyrB*, *rpoB* gene sequences of isolate WS05 (GenBank accession nos. MN148709, MN394631, MN394672) respectively showed similarities of 99% with the known strains of *A. hydrophila* in the GenBank database. Phylogenetic trees constructed by the neighbor-joining method demonstrated isolate WS05 to be an *A. hydrophila* strain (Figure 1, Figure 6 and Figure 7), which is in agreement with the phenotypic identification of isolate WS05 (Table 1).

### 3.3. Antibiotic Susceptibility of the Pathogenic Isolate

The data (Table S1) indicated that isolate WS05 was susceptible to a range of veterinary antibiotics except novobiocin and rifampicin, including amoxicillin, cotrimoxazole, cefotaxime, doxycycline, enrofloxacin, florfenicol, gentamicin, kanamycin, nalidixic acid, neomycin, netilmicin, oxacillin, polymyxin B, streptomycin, tetracycline, and tobramycin. In particular, isolate WS05 apparently exhibited susceptibility to cotrimoxazole, doxycycline, florfenicol, neomycin, and tetracycline, which are currently approved for use in aquaculture [53].

### 3.4. Synergistic Effect of Florfenicol–Herb Extracts

The MICs of florfenicol and herb extracts alone or in combination against isolate WS05 are presented in Table 2. The result indicated that only the combination of florfenicol and *P. granatum* extract had a synergistic antibacterial effect on isolate WS05. When combined with 7.81 mg mL^−1^ *P. granatum* extract, the MIC of florfenicol against isolate WS05 was reduced from 0.50 to 0.03 mg L^−1^. The FICI for the combination of florfenicol and *P. granatum* extract was calculated as 0.31. However, florfenicol exhibited additive effects on isolate WS05 when combined with extracts from *A. argyi, A. capillaries, Galla chinensis*, *P. mume*, *Radix aucklandiae*, and *Radix sophorae flavescentis*. The joint effects of florfenicol with extracts from other herbs (*C. segetum, F. toosendan*, *P. aviculare*, *Polygonum cuspidatum*, *Radix et rhizoma rhei*, *Radix scutellariae*, *Radix sanguisorbae*, *Radix bupleuri*, *Rhizoma acori tatarinowii*) showed no interactions. This suggests that *P. granatum* extract can be considered as a potential synergist of florfenicol against isolate WS05.

### 3.5. The in Vitro Activity of Florfenicol-P. granatum Extract

**Figure 8:**
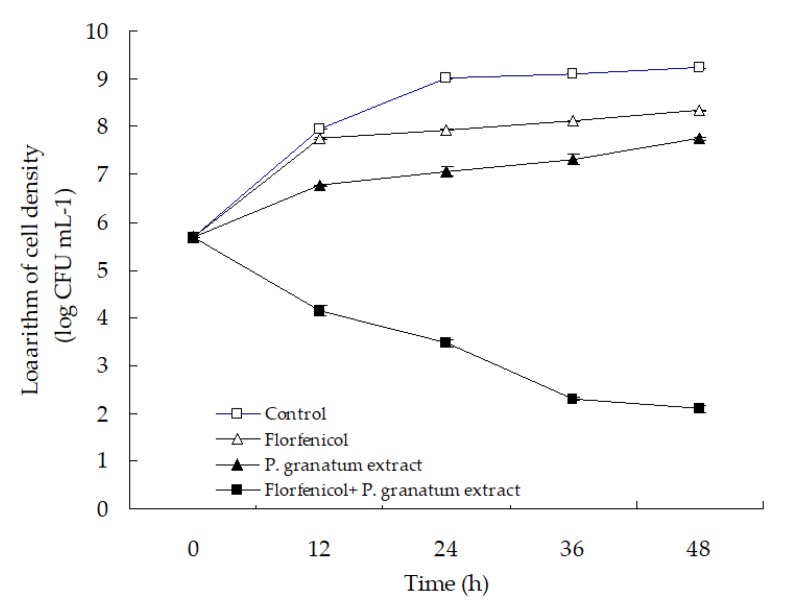
The time-kill curve with 0.03 mg L^−1^ florfenicol and 7.81 mg mL^−1^ *P. granatum* extract alone or in combination against isolate WS05.

The time–kill curve with florfenicol and *P. granatum* extract alone or in combination is presented in Figure 8. No reduction in the cell density of isolate WS05 was observed in the control group, and the single treatment with florfenicol and *P. granatum* extract only showed a slight antibacterial activity in comparison with the control. In contrast, the combination of florfenicol and *P. granatum* extract exhibited a significant antibacterial activity against isolate WS05, with a significant reduction of ≥3.61 log in cell density after 24 h of treatment compared with that in the single antimicrobial treatment (*p* < 0.05). According to the criteria suggested by Pillai et al. (2005) [54] that synergism is defined as a respective decrease of ≥2 log CFU mL^−1^ in antimicrobial activity produced by the combination compared with that by the more active agent alone after 24 h, the in vitro antibacterial activity of the combination of florfenicol and *P. granatum* extract can be categorized as synergism, which is in line with the findings in the synergistic effect assay above.

### 3.6. Protective Effect of Florfenicol-P. granatum Extract

**Figure 9:**
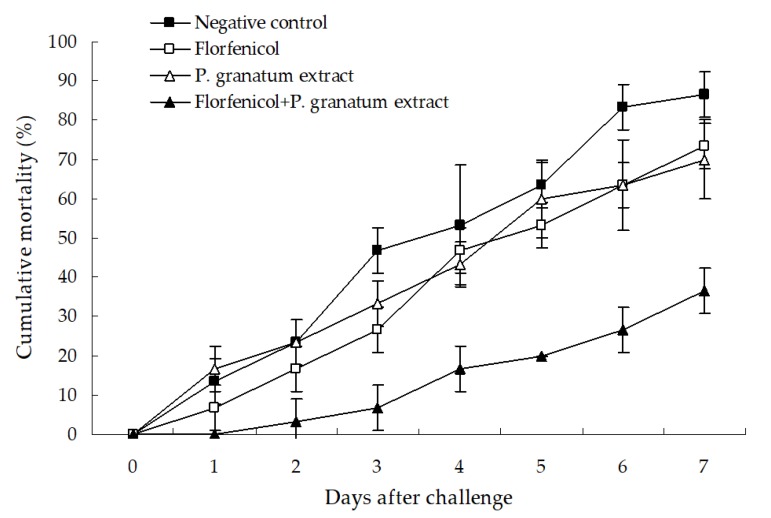
Protection of 0.03 mg L^−1^ florfenicol and 7.81 mg mL^−1^ *P. granatum* extract alone or in combination against the challenge of isolate WS05 in whiteleg shrimp. Data are presented as the mean ± SD.

The protective effect of florfenicol and *P. granatum* extract alone or in combination is shown in Figure 9. No mortality was observed in the test whiteleg shrimp in the blank and positive control groups (data not shown), and the single treatment with florfenicol and *P. granatum* extract only showed a slightly protective effect in comparison with the negative control, with only a 13.34% and 16.67% decrease in the cumulative mortality of shrimp as compared with the negative control. However, a significant protection was produced by the combination of florfenicol and *P. granatum* extract, with a cumulative mortality of 36.66% (*p* < 0.05) and 33.33% (*p* < 0.05) lower than that in the single treatment with florfenicol and *P. granatum* extract after the challenge with isolate WS05 for seven days. The death of all of the test shrimp was caused by the challenge strains, as determined by bacterial isolation and identification (data not shown). These findings indicated that the combination of florfenicol and *P. granatum* extract could potentiate the protective effect against *A. hydrophila* infection in freshwater-farmed whiteleg shrimp.

## 4. Discussion

The impact of *A. hydrophila* in aquaculture has been well documented, with massive mortality reported in fairy shrimp *Branchipus schaefferi* (Fisher), crayfish *Pacifastacus leniusculus*, climbing perch *Anabas testudineus*, grass carp *Ctenopharynngodon idellus*, silver carp *Hypophthalmichthys molitrix*, southern catfish *Silurus meridionalis*, white bream *Parabramis pekinensis*, and Murray cod *Maccullochella peelii* [55,56,57,58,59,60,61,62]. However, there is limited information on *A. hydrophila* infection in freshwater-cultured whiteleg shrimp. Therefore, more attention should be given for whiteleg shrimp–pathogenic *A. hydrophila*. 

Multiple virulence factors have been demonstrated to play an important role in the pathogenicity of *A. hydrophila*, including flagella, elastase, haemolysins, enterotoxins, and aerolysin [63,64,65,66,67]. Diseases caused by *A. hydrophila* in aquaculture are usually associated with the production of these virulent factors [68]. In general, the isolated bacteria in aquaculture were classified as virulent, weakly virulent, or avirulent based on the dose and survival time after challenge [56]. A virulent *Vibrio parahaemolyticus* strain VP1 isolated from vibriosis-infected penaeid shrimp in India was reported to kill 35% of shrimp after 7 days of bath challenge at final concentrations of 2.1 × 10^4^ to 1.3 × 10^5^ CFU mL^−1^ [69]. Our data indicated that isolate WS05 of *A. hydrophila* could cause an average mortality of 40% to 55% mortality in healthy shrimp after 7 days of challenge at 2.0 × 10^4^ to 2.0 × 10^5^ CFU mL^−1^, with an LD_50_ value of 4.8 × 10^4^ CFU mL^−1^. This reveals that *A. hydrophila* isolate WS05 is more virulent than *V. parahaemolyticus* VP1 and can pose a more serious threat to penaeid shrimp farming. In addition to the virulence of *A. hydrophila* isolate WS05, there might be other secondary factors that induce this disease, which should also be raised as concerns, such as ammonia and sulfide, which can increase the susceptibility of whiteleg shrimp to bacterial infections by depression of immune capability [70,71].

In many cases, *A. hydrophila* is abundant in water fauna and is commonly associated with copepods, which can further transport this bacterium to other farming regions to result in aeromonasis [56]. Therefore, *A. hydrophila* isolate WS05 may constitute a danger for many *Aeromonas*-susceptible aquatic animals and the control of this isolate should not be underestimated. Several studies have reported that over 300 strains of *A. hydrophila* from aquatic animals have exhibited a relatively high resistance to rifampicin and novobiocin [72,73]. This is also observed in *A. hydrophila* isolate WS05. In addition, our data indicated that *A. hydrophila* isolate WS05 was susceptible to cotrimoxazole, doxycycline, florfenicol, neomycin, and tetracycline, which can be used in the treatment of this disease. However, one of the most important problems involving treatments with antibiotics against *A. hydrophila* in aquaculture is that antibiotic resistance develops readily [74]. Thus, new techniques to prevent antibiotic resistance development in pathogenic *A. hydrophila* are needed to make the aquaculture industry more sustainable [75].

Currently, an effective approach to overcome the resistance of bacterial pathogens is a combination of herb extracts with antibiotics. Lee et al. (1998) found that combinations of chloramphenicol and extracts from several herbs, such as *Acanthopanax koreanum*, *Artemisia iwayomogi*, *Clerodendron trichotomum*, *Juniperus rigida*, *Magnolia sieboldii*, *Pinus densiflora*, *Pinus koraiensis*, and *Zanthoxylum piperitum*, had resistance inhibitory activities against multidrug-resistant *Staphylococcus aureus* [76]; Liu et al. (2010) demonstrated that baicalin isolated from *Scutellaria amoena* had the potential to restore the effectiveness of β-lactam antibiotics against β-lactam-resistant *Staphylococcus aureus* and could potentiate the killing of β-lactam-resistant *S. aureus* cells [49]; and Haroun and Al-Kayali (2006) confirmed that *Thymbra spicata* extracts could significantly increase the antibacterial action of cefotaxime against multidrug-resistant *S. aureus* and *Klebsiella pneumoniae* strains [50]. In our study, *P. granatum* extract potentiated the antimicrobial action of florfenicol, suggesting a possible utilization of this herb extract in combination with florfenicol against *A. hydrophila* pathogens because a large majority of *A. hydrophila* strains are susceptible to phenicol antibiotics, like florfenicol [72,73]. Similar findings were also made by Endo et al. (2010) [77], in that a combination of *P. granatum* extract and fluconazole has a synergistic action against fungal pathogens. Besides its antibacterial activity [78], the synergistic activity of *P. granatum* extract may be attributed to the ability of its active component, like punicalagin, gallic acid, and chlorogenic acid, to disturb the cell wall and depolarize the cytoplasmic membrane and then facilitate the influx of florfenicol inside bacterial cells to strongly inhibit bacterial protein synthesis [79,80,81].

In conclusion, for the first time, the present study identified *A. hydrophila* as a causative agent of whiteleg shrimp, and indicated that *P. granatum* extract potentiated the antimicrobial action of florfenicol, suggesting a possible utilization of this herb in combination with florfenicol against shrimp-pathogenic *A. hydrophila*.

## Figures and Tables

**Table 1:** Phenotypic characterization of isolate WS05.

Tests	Reaction	
	Isolate WS05	*A. hydrophila* ^a^
Arginine dihydrolase	R ^+^	R ^+^
Cytochrome oxidase	R ^+^	R ^+^
β-Galactosidase	R ^+^	R ^+^
Gelatinase	R ^+^	R ^+^
Lysine decarboxylase	R ^+^	R ^+^
Ornithine decarboxylase	R ^−^	R ^−^
Tryptophan deaminase	R ^−^	R ^−^
Urease	R ^−^	R ^−^
Citrate utilization	R ^+^	R ^+^
Acetoin production	R ^+^	R ^+^
Indole production	R ^+^	R ^+^
H_2_S production	R ^−^	R ^−^
Arabinose fermentation	R ^+^	R ^+^
Amygdalin fermentation	R ^−^	R ^−^
Glucose fermentation	R ^+^	R ^+^
Inositol fermentation	R ^−^	R ^−^
Mannitol fermentation	R ^+^	R ^+^
Melibiose fermentation	R ^−^	R ^−^
Rhamnose fermentation	R ^−^	R ^−^
Sucrose fermentation	R ^+^	R ^+^
Sorbitol fermentation	R ^−^	R ^−^

R ^+^. Positive reaction; R ^−^. Negative reaction; a. The reference strains of *A. hydrophila* reported by Long et al. (2016) [41] and Ye et al. (2018) [42].

**Table 2:** MICs of florfenicol and herb extracts alone or in combination against isolate WS05.

Drug Alone	MICs	Drug Combination	MICs in Combination		FICI
			FFC	Herb Extract	
FFC	0.50 ± 0 ^h^	FFC + AA	0.13 ± 0 ^d^	15.63 ± 0 ^e^	0.75 ± 0 ^fg^
AA	31.25 ± 0 ^e^	FFC + AC	0.03 ± 0 ^e^	62.5 ± 0 ^b^	0.56 ± 0 ^gh^
AC	125 ± 0 ^b^	FFC + RAT	0.5 ± 0 ^b^	15.63 ± 0 ^e^	1.06 ± 0 ^def^
RAT	250 ± 0 ^a^	FFC + CS	0.03 ± 0 ^e^	62.5 ± 0 ^b^	1.06 ± 0 ^def^
CS	62.5 ± 0 ^c^	FFC + FT	1.00 ± 0 ^a^	5.86 ± 2.76 ^ef^	2.19 ± 0.09 ^a^
FT	31.25 ± 0 ^e^	FFC + GC	0.21 ± 0.06 ^c^	2.60 ± 0.92 ^f^	0.67 ± 0.12 ^gh^
GC	10.41 ± 3.68 ^g^	FFC + PM	0.03 ± 0 ^e^	7.81 ± 0 ^ef^	0.89 ± 0.24 ^efg^
PM	10.41 ± 3.68 ^g^	FFC + PA	0.5 ± 0 ^b^	52.08 ± 14.73 ^c^	1.42 ± 0.12 ^cd^
PA	125 ± 0 ^b^	FFC + PG	0.03 ± 0 ^e^	7.81 ± 0 ^ef^	0.31 ± 0 ^h^
PG	31.25 ± 0 ^e^	FFC + PC	1.00 ± 0 ^a^	15.63 ± 0 ^e^	2.06 ± 0 ^ab^
PC	250 ± 0 ^a^	FFC + RRR	0.21 ± 0.06 ^c^	31.25 ± 0 ^d^	1.25 ± 0.2 ^de^
RRR	41.67 ± 14.73 ^d^	FFC + RA	0.13 ± 0 ^d^	26.04 ± 7.36 ^d^	0.67 ± 0.12 ^gh^
RA	62.5 ± 0 ^c^	FFC + RS	0.03 ± 0 ^e^	31.25 ± 0 ^d^	1.73 ± 0.47 ^bc^
RS	20.84 ± 7.36 ^f^	FFC + RSF	0.03 ± 0 ^e^	62.5 ± 0 ^b^	0.56 ± 0 ^gh^
RSF	125 ± 0 ^b^	FFC + RSA	0.21 ± 0.06 ^c^	11.72 ± 5.52 ^ef^	1.08 ± 0.24 ^def^
RSA	20.84 ± 7.36 ^f^	FFC + RB	0.03 ± 0 ^e^	500 ± 0 ^a^	2.06 ± 0 ^ab^
RB	250 ± 0 ^a^				

MIC_S_. Minimum inhibitory concentrations; FFC. Florfenicol; AA. *Artemisia argyi*; AC. *Artemisia capillaries*; RAT. *Rhizoma acori tatarinowii*; CS. *Cephalanoplos segetum*; FT. *Fructus toosendan*; GC. *Galla chinensis*; PM. *Prunus mume*; PA. *Polygonum aviculare*; PG. *Punica granatum*; PC. *Polygonum cuspidatum*; RRR. *Radix et rhizoma rhei*; RA. *Radix aucklandiae*; RSC. *Radix scutellariae*; RSF. *Radix sophorae flavescentis*; RSA. *Radix sanguisorbae*; RB. *Radix bupleuri*. Data are presented as the mean ± SD with mg L^−1^ for florfenicol and mg mL^−1^ for herb extracts. Values with different superscript letters in the same column indicate statistically significant difference (*p* < 0.05).

## Supplementary Materials

The following are available online at https://www.mdpi.com/2076-2607/7/10/450/s1.
