# Supplementary material for: Aeromonas hydrophila, an Emerging Causative Agent of Freshwater-Farmed Whiteleg shrimp Litopenaeus vannamei
Source: Microorganisms. 2019 Oct 14;7(10):450. doi: 10.3390/microorganisms7100450 (PMC6843590; doi:10.3390/microorganisms7100450)
Supplement: Supplementary file 1 [file microorganisms-07-00450-s001.pdf]

Supplementary Materials

Aeromonas hydrophila, an Emerging Causative Agent of Freshwater-farmed Whiteleg shrimp *Litopenaeus vannamei*

Huihua Zhou[1], Chunlei Gai[2], Guifang Ye[1], Jian An[3], Kai Liu[4], La Xu[2] and Haipeng Cao[1,*]

[1] National Pathogen Collection Center for Aquatic Animals, Shanghai Collaborative Innovation for Aquatic Animal Genetics and Breeding, Shanghai Engineering Research Center of Aquaculture, Shanghai Ocean University, Shanghai 201306, P.R. China.

[2] Marine Biology Institute of Shandong, Qingdao Shandong 266104, P.R. China.

[3] Lianyungang Marine and Fisheries Development Promotion Center, Lianyungang Jiangsu 222000, P.R. China.

[4] Institute of Fishery Science, Hangzhou Academy of Agricultural Sciences, Hangzhou Zhejiang 310024, P.R. China.

The first two authors contributed equally to this work.

* Correspondence: e-mail: hpcao@shou.edu.cn; Tel: +862161900453; Fax: +862161900452.

Table S1. Susceptibility of isolate WS05 to veterinary antibiotics.

Antibiotics	Content (μg/disc)	Inhibition zone diameter (mm)
Amoxicillin	10	20.3±0.5[S]
Cotrimoxazole	23.75/1.25	23.3±0.9[S]
Cefotaxime	30	46.7±0.5[S]
Doxycycline*	30	23.7±0.5[S]
Enrofloxacin*	5	34.3±0.5[S]
Florfenicol*	30	35.0±0.8[S]
Gentamicin	10	28.7±0.5[S]
Kanamycin	30	25.3±0.5[S]
Nalidixic acid	30	34.3±0.5[S]
Neomycin*	30	23.7±0.5[S]
Netilmicin	30	28.3±0.5[S]
Novobiocin	30	9.0±0.8[R]
Oxacillin	1	18.3±0.5[S]
Polymyxin B	30	15.3±0.5[S]
Rifampicin	5	11.0±0[R]
Streptomycin	10	24.3±0.5[S]
Tetracycline*	30	26.3±0.5[S]
Tobramycin	10	27.3±0.5[S]

Data are presented as the mean ± standard deviation of three independent experiments. [S]Sensitive; [R]Resistant.*Veterinary antibiotics used in aquaculture.

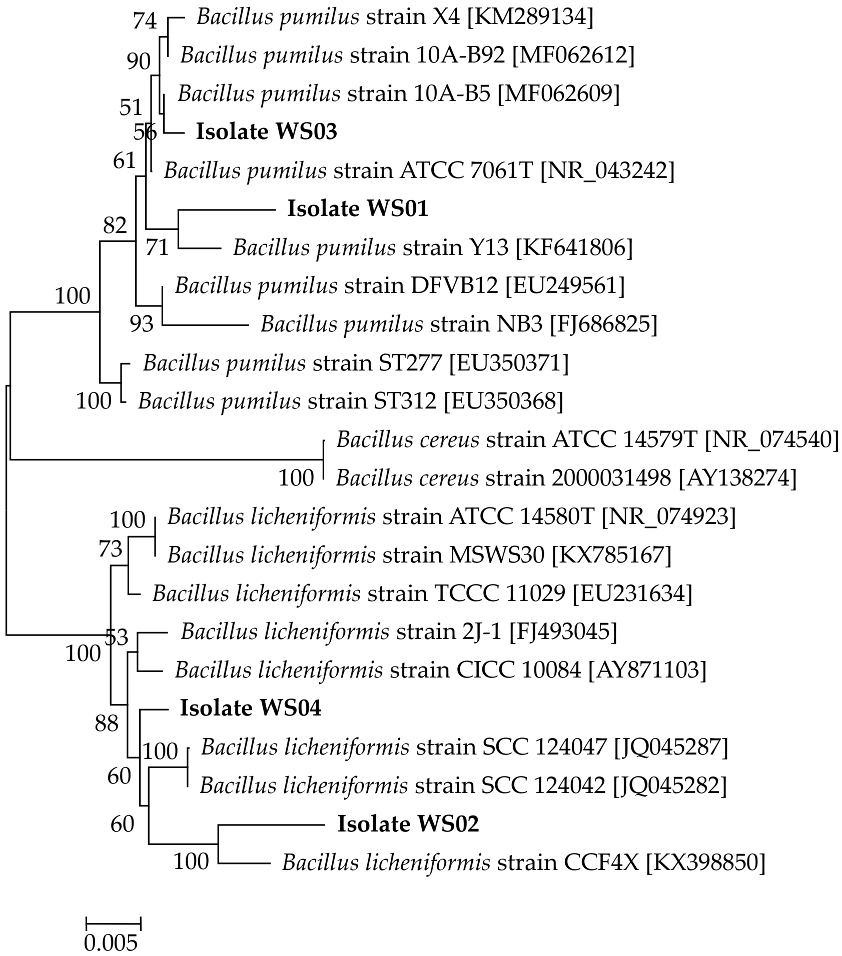

Figure S1. The 16S rRNA phylogenetic tree of 19 known bacteria and isolates WS01, WS02, WS03 and WS04 constructed using neighbor-joining method. The length of aligned sequences is 1207 bp. The bootstrap values (%) are shown besides the clades, accession numbers are indicated beside the name of strains, and scale bars represent distance values.
